# Unmasking Hidden Clues in Cutaneous Viral Infections: Histopathological Insights Into Keratinocytic Features of Herpes Simplex Virus, Varicella Zoster Virus, and Molluscum Contagiosum

**DOI:** 10.7759/cureus.98882

**Published:** 2025-12-10

**Authors:** Tania Platero Portillo, Dilshad Dhaliwal, Sepideh Mehravaran, Megan Ketcham, Julie Wu, Nisha Ramani, Abdul Hafeez Diwan

**Affiliations:** 1 Department of Pathology and Immunology, Baylor College of Medicine, Houston, USA; 2 Department of Pathology, Moffitt Cancer Center, Tampa, USA; 3 Department of Pathology, Houston Methodist Hospital, Houston, USA; 4 Ronald O. Perelman Department of Dermatology, New York University Grossman School of Medicine, New York, USA

**Keywords:** cutaneous viral infections, hsv, molluscum contagiosum, viral infections of the skin, vzv

## Abstract

Objective: This case study aimed to explore primarily the keratinocytic changes of cutaneous viral infections caused by herpes simplex virus (HSV), varicella-zoster virus (VZV), and molluscum contagiosum (MC). The focus was on identifying features and diagnostic clues in cases without typical findings.

Methods: A retrospective analysis encompassing 14 HSV/VZV cases (January 2000 to October 2023) and 22 MC cases (January 2013 to December 2015) was conducted.

Results: In HSV/VZV cases, 71% (10 of 14) exhibited distinctive histopathological features in the adjacent keratinocytes, including nucleolar enlargement and atypical features. Immunohistochemical studies in 40% of the cases (4 of 10) revealed positive staining in cells with cytopathic changes and negative staining in adjacent atypical keratinocytes. Conversely, MC cases consistently demonstrate an enlarged, stuffed "pregnant seahorse appearance" of keratinocytes around molluscum bodies.

Conclusion: This study highlights the diagnostic significance of keratinocytic changes in these viral infections, allowing pathologists to perform additional studies to make the correct diagnosis in cases where typical viral changes are not readily apparent.

## Introduction

Dermatological manifestations of viral infections, particularly those caused by herpes simplex virus (HSV), varicella-zoster virus (VZV), and molluscum contagiosum (MC), often present with characteristic clinical features that aid in their diagnosis [1, 2]. For HSV/VZV infections, it is well-documented that specific patient groups, such as those with compromised immune systems, may exhibit atypical presentations, posing challenges that require supplementary testing and histopathological examination for precise diagnosis [3, 4]. Skin biopsies of HSV/VZV infections reveal comparable histopathological findings, making it impossible to definitively distinguish between HSV and VZV infections based on morphology alone. Pathologic features include multinucleation, nuclei with a ground-glass or steel-gray appearance, eosinophilic intranuclear inclusions (Cowdry A bodies), marginated chromatin [1, 3, 5], and the presence of eosinophils [6]. Although typically straightforward, the confirmatory features of cutaneous HSV/VZV infection may not consistently appear in biopsy specimens.

The causative agent of MC infection is the molluscum contagiosum virus (MCV), a double-stranded DNA virus belonging to the* Poxviridae* family, exclusive to humans as hosts. MCV infects the epidermis and undergoes replication in the cytoplasm of cells, with an incubation period ranging between two and six weeks [7, 8]. MC is a naturally resolving infectious dermatosis that commonly affects the pediatric population, sexually active adults, and immunocompromised individuals [9]. The clinical diagnosis of MC relies on the identification of distinct umbilicated papules. The clinical differential diagnoses vary based on age and immunological status, with histoplasmosis and cryptococcosis being key considerations in immunosuppressed patients, both potentially presenting as umbilicated papules [7, 9]. Histopathological examination reveals characteristic intracytoplasmic eosinophilic inclusion bodies known as Henderson-Patterson bodies [10].

This case study explored the keratinocytic features observed in cutaneous infections caused by HSV, VZV, and MC. These insights not only contribute to a more nuanced understanding of viral dermatoses but also have practical implications for diagnostic approaches in challenging cases.

## Materials and methods

Materials and methods

This study was a retrospective analysis conducted at the Department of Pathology and Immunology at Baylor College of Medicine. It was approved by the Institutional Review Board (IRB) under umbrella protocols ESP1:H-24175 and H47540. The study aimed to evaluate histopathological features of cutaneous viral infections, including HSV, VZV, and MC.

Study design and data collection

A comprehensive search of the institutional pathology information system (Vista database) was performed for all cases diagnosed as HSV or VZV infection between January 2000 and October 2023, and sequential MC cases diagnosed between January 2013 and December 2015. Search terms included "herpes," "HSV," "VZV," "varicella," and "molluscum." Cases were included if they had histopathological confirmation of infection based on hematoxylin and eosin (H&E) staining and ancillary diagnostic testing (e.g., immunohistochemistry). Exclusion criteria included incomplete clinical data, missing slides, or inadequate tissue for review.

For HSV and VZV cases, the following data were collected: patient demographics (age and gender), biopsy site, results of ancillary diagnostic testing (e.g., immunohistochemical studies), and histopathological features observed on H&E-stained slides. For MC cases, patient demographics, biopsy site, and histopathological findings were reviewed. Two cases of MC that were submitted for consultation due to concerns about atypia were also included.

Slide review and histopathological analysis

H&E-stained slides were retrieved for each case and reviewed independently by two board-certified pathologists: one dermatopathologist (DD) and one surgical pathologist (TPP). Consensus was reached in cases with discrepancies. A standardized evaluation form was used to document features such as viral cytopathic effects (e.g., multinucleation, ground-glass nuclei, Cowdry A inclusions, ballooning degeneration), nucleolar enlargement, chromatin margination, and keratinocytic atypia.

For HSV and VZV cases, histopathological features of keratinocytes and surrounding stroma were analyzed. For MC cases, emphasis was placed on identifying keratinocytic atypia adjacent to molluscum bodies (MBs), including prominent nucleoli, amphophilic cytoplasm, and surrounding stromal changes.

Immunohistochemistry 

Immunohistochemical stains for HSV-1 and HSV-2 were reviewed when available (four cases). Staining was performed on formalin-fixed, paraffin-embedded tissue sections using an automated immunostainer according to the manufacturer’s protocol. Nuclear staining in keratinocytes with cytopathic changes was interpreted as positive. Keratinocytes with enlarged, atypical nuclei lacking HSV-1/2 staining were recorded as negative.

Diagnostic criteria

HSV and VZV infections were diagnosed based on hallmark features, including multinucleation, ground-glass nuclei, Cowdry A inclusions, and ballooning degeneration. MC infections were diagnosed based on the presence of Henderson-Patterson bodies and characteristic keratinocytic changes. Deeper levels were performed when initial sections were inconclusive, particularly in cases referred for atypia.

Statistical analysis

The data were analyzed descriptively, with categorical variables expressed as frequencies (n) and percentages (%). Statistical software (SPSS version 25, IBM Corp., Armonk, NY) was used for calculations. No interobserver agreement or hypothesis testing was performed due to the descriptive and exploratory nature of the study.

## Results

HSV/VZV cases

In 71% of these cases (10 of 14), keratinocytes adjacent to cells displaying clear viral cytopathic changes exhibited significant nucleolar enlargement and atypical features. These keratinocytes were characterized by an abundance of amphophilic cytoplasm surrounding large, pale, and round nuclei with consistently clear chromatin displaying early margination. Some of these cells also exhibited nucleoli that were pushed toward the periphery.

**Figure 1:**
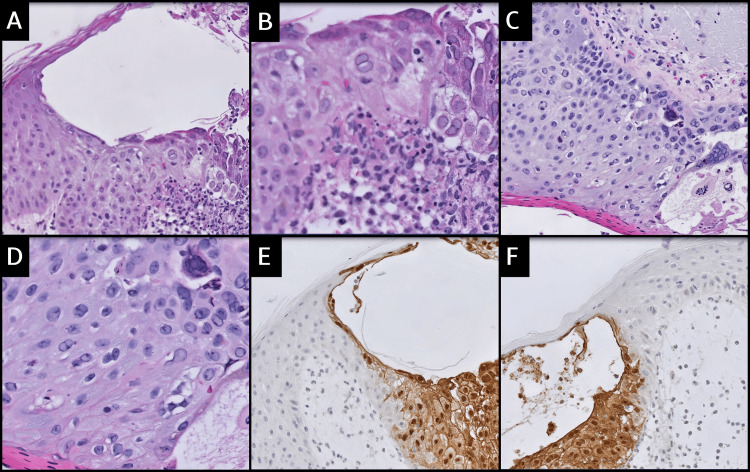
A-D. Significant nucleolar enlargement and atypical features in keratinocytes adjacent to cells with viral cytopathic changes (A 200x, B 400x, C 200x, D 200x). E-F. HSV 1-2 immunohistochemical stain displays positive staining in cells with characteristic cytopathic changes, along with negative staining in adjacent atypical keratinocytes (E 200x, F 200x).

In 40% of the cases (4 of 10) in which these changes were observed, a previously performed immunohistochemical study of HSV-I and HSV-II revealed positive staining of cells exhibiting characteristic cytopathic changes, while simultaneously showing negative staining in adjacent atypical keratinocytes. The stroma adjacent to the herpes virus showed edematous or fibromyxoid changes in 100% of the cases (14 of 14). Additionally, 57% (8 of 14) had associated abundant inflammation, and 35% (5 of 14) had an ulcerated background in addition to stromal changes. Hyperchromatic nuclei and atypical mitoses were not observed in any of the cases. Figure 1A-1D visually exemplifies these findings, emphasizing the significant nucleolar enlargement and atypical features in keratinocytes adjacent to cells with viral cytopathic changes. In Figure 1E-1F, HSV 1-2 immunohistochemical stain displays positive staining in cells with characteristic cytopathic changes, along with negative staining in adjacent atypical keratinocytes, emphasizing the pivotal role of recognizing atypical features for an accurate diagnosis.

In all the cases we reviewed, we consistently observed hallmark histopathological features associated with herpetic infections, including acantholysis, ballooning degeneration, intranuclear inclusions, multinucleation, and necrosis, with some cases showing the development of vesicles or ulcers. However, in 78% of these cases (11 of 14), the diagnostic findings were not readily identified, prompting the use of levels or ancillary studies to confirm the diagnosis. Table 1 summarizes the histopathological characteristics observed in patients with cutaneous HSV/VZV infection. Among the 14 patients included in this study, 71% (10 of 14) exhibited atypical keratinocytes adjacent to cells with viral cytopathic changes (as noted above). Ulceration was present in 35% of the cases (5 of 14). VZV/HSV immunohistochemical studies were performed in 29% of the cases (4 of 14). This table provides a quantitative overview of these histopathological features, offering insights into the patterns observed in the retrospective analysis.

**Table 1:** Histopathologic characteristics of examined cases of cutaneous HSV/VZV infection. VZV: varicella-zoster virus; HSV: herpes simplex virus; IHC: Immunohistochemistry

Histologic and Immunohistochemical Features	Total (n = 14)	Percentage
Atypical keratinocytes adjacent to cells with viral cytopathic changes	10	71%
Ulceration		
Present	5	36%
Absent	9	64%
VZV/HSV IHC		
Performed	4	29%
Not performed	10	71%

MC cases

**Figure 2:**
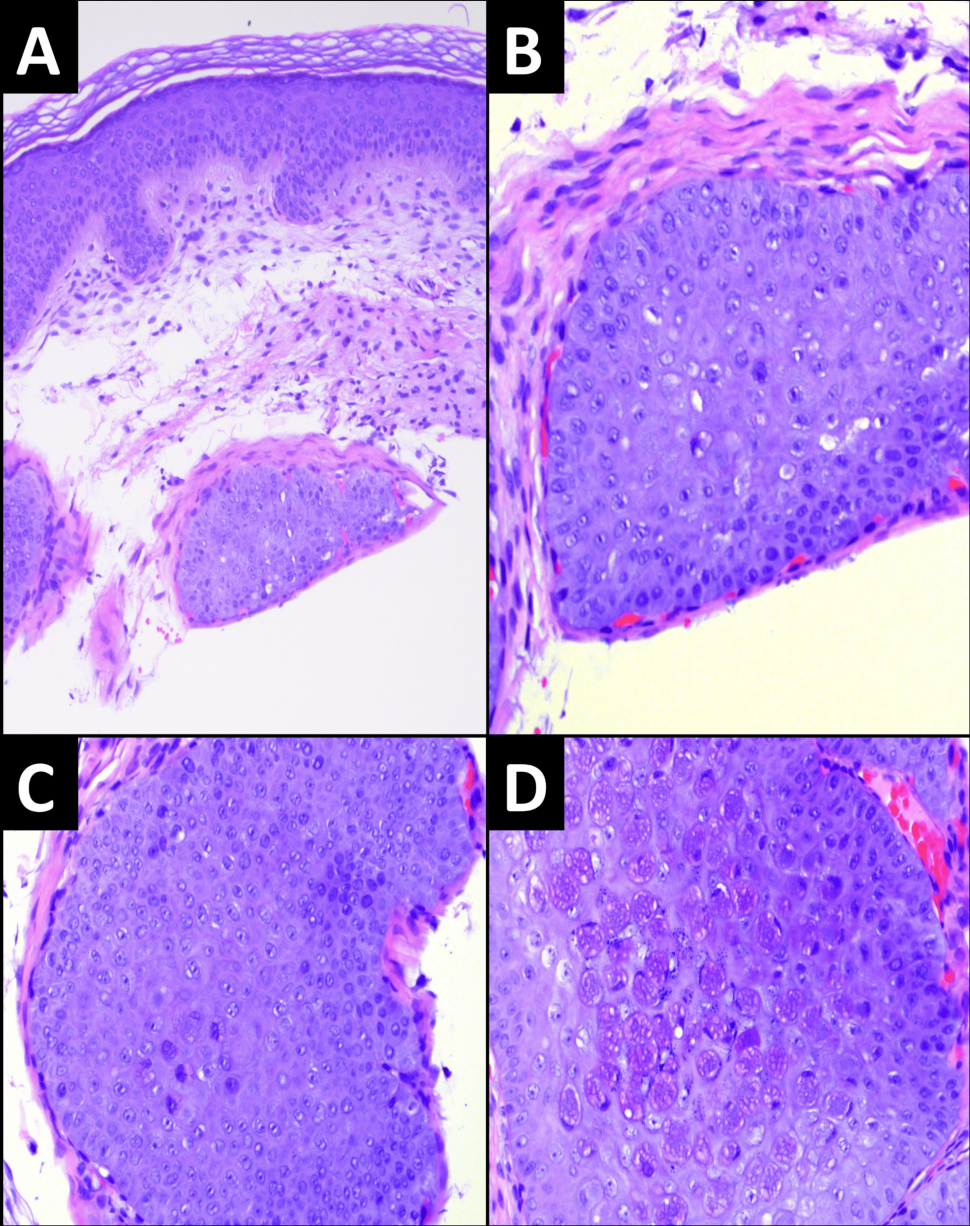
A-C. Enlarged keratinocytes in initial sections, in the absence of diagnostic MB (A 100x, B 400x, C 200x). Higher power sections show prominent nucleoli and amphophilic cytoplasm. D. Deeper sections in the block show well-formed MBs are present within the central portion of the lesion (400x).

A retrospective review of MC cases revealed that, in 100% of cases (22 of 22), the keratinocytes adjacent to the cells with obvious molluscum bodies (MBs) had large, prominent nucleoli and atypia. These atypical features were precisely those that prompted consultation. These keratinocytes had amphophilic cytoplasm surrounding the atypical nuclei. The stroma adjacent to the MC showed edematous or fibromyxoid changes in 59% (13 of 22) of patients. A few cases (23%, 5 of 22) had associated inflammation in addition to stromal changes. Hyperchromatic nuclei and atypical mitoses were not observed in any of the cases. An example of a case that was sent in consultation for the evaluation of squamous atypia is shown in Figure 2. Note the “stuffed” appearance of keratinocytes on the initial sections without any MBs (Figure 2A-2C) and around the MBs (Figure 2D).

## Discussion

Cutaneous eruptions caused by herpes simplex 1/2 (HSV-1/2) and HSV/VZV are common dermatoses. Typically, a correct diagnosis relies on characteristic clinical findings, with skin lesions biopsied only in cases with unusual presentations [4, 11]. Alpha herpesviruses, including HSV and VZV, have a distinct tendency to establish latent, lifelong infections in the dorsal root sensory ganglia, periodically reactivating to cause asymptomatic viral shedding or clinical disease. These characteristics significantly influence the clinical manifestations of HSV and VZV [3, 12].

Both viruses create intraepidermal vesicles, with varying degrees of epithelial necrosis. Typical histopathological findings include keratinocytes displaying ballooned nuclei with a ground-glass appearance and giant multinucleated keratinocytes. HSV and VZV are known for characteristic features such as acantholysis, ballooning degeneration, intranuclear inclusions, multinucleation, necrosis, and vesicle or ulcer formation [3, 8, 13]. In immunosuppressed or hematologically compromised individuals, cutaneous herpes infections often present atypically, posing diagnostic challenges. Differential diagnosis includes cutaneous lymphoma or pseudolymphoma [12, 14]. Histological variations in biopsied cases range from pure epithelial lesions to lesions resembling malignant lymphoma with a pseudolymphomatous pattern [12, 14, 15]. In our study, we emphasize a distinctive aspect of atypical features in neighboring keratinocytes, serving as a diagnostic indicator, warranting additional investigation. Notably, we highlighted the unique keratinocytes adjacent to the cells displaying evident herpetic changes in routine H&E-stained sections. Features include nucleolar enlargement, atypical features, and early margination changes, presenting diagnostic clues that are particularly beneficial in challenging cases to differentiate HSV/VZV from other diagnoses. Moreover, it is noteworthy that in 40% of the observed cases (4 of 10), HSV-I and HSV-II immunohistochemical studies demonstrated positive staining of cells displaying characteristic cytopathic changes coupled with concurrent negative staining of adjacent atypical keratinocytes.

MC results in persistent localized skin infections that can manifest in various parts of the body [2, 16]. Individuals with compromised cellular immunity are at an elevated risk of developing severe and challenging infections. The differential diagnosis of MC encompasses conditions such as cryptococcosis, histoplasmosis, basal cell carcinoma, and warts [7, 9].

In 2007, Calder et al. [17] reported noteworthy changes in keratinocytes adjacent to those containing characteristic molluscum bodies (MB). In their study, the authors emphasized the violaceous appearance of keratinocytes surrounding the MB. Subsequently, in 2015, Ishikawa et al. [18] contributed significantly to the understanding of MC by exploring the histopathological features beyond MB. This study is particularly noteworthy because MB may not be readily identifiable in superficial sections. Ishikawa et al. identified crucial features, including prominent nucleoli; amphophilic cytoplasm; clear cytoplasmic vacuolization; and edematous, fibromyxoid, and mucinous stroma [18].

Retrospective review of MC cases revealed a consistent feature we call the "pregnant seahorse appearance" in keratinocytes around molluscum bodies, given the resemblance of the "stuffed" keratinocytes to pregnant seahorses. This distinctive feature, characterized by enlarged, stuffed keratinocytes with violaceous or amphophilic coloration, prompted consultation in cases that were initially suspected of atypia. Deeper sections consistently revealed characteristic molluscum bodies.

We hypothesize that this appearance may reflect virus-induced alterations in keratinocyte maturation or cytoskeletal remodeling. Further ultrastructural or molecular studies may help clarify the pathophysiology of this phenomenon.

This study has several strengths. First, it addresses a practical diagnostic challenge in dermatopathology by highlighting underrecognized keratinocytic changes that may aid in identifying viral infections when classical features are absent. Second, it incorporates cases from two different viral families (herpesviridae and poxviridae), allowing for comparison of shared reactive keratinocytic patterns. Third, the study leverages immunohistochemistry in HSV/VZV cases to distinguish between cytopathic and adjacent reactive keratinocytes, reinforcing the diagnostic value of these patterns.

This is analogous to pregnant seahorses giving birth, with deeper sections unveiling the characteristic molluscum bodies. In most cases, the stroma adjacent to the MC displayed edematous or fibromyxoid changes in most cases, with some showing associated inflammation.

The diagnostic clues identified in both HSV/VZV and MC cases have significant implications in histopathological practice. Recognition of these distinctive features in adjacent keratinocytes not only serves as a valuable indicator for pathologists but also prompts further investigation, particularly in clinically ambiguous or histologically subtle presentations.

However, due to the retrospective nature of this study, the lack of control groups, and the absence of blinded assessments or interobserver reproducibility data, our findings should be interpreted as descriptive and exploratory.

No diagnostic accuracy metrics (e.g., sensitivity or specificity) were calculated, and the diagnostic performance of these keratinocytic features remains to be validated. Despite these limitations, the observed patterns provide practical insight that may inform future diagnostic approaches in dermatopathology.

Study limitations 

Despite the valuable insights provided by this study, several limitations must be acknowledged. First, the retrospective design inherently introduces selection and information biases, as it relies on existing records and slide availability. This limitation was evident in the small sample size of HSV/VZV cases, where missing slides and incomplete data reduced the number of evaluable cases to 14. Furthermore, the study was conducted at a single institution, which may limit the generalizability of the findings to broader populations. Another limitation is the reliance on ancillary studies and deeper sections to confirm diagnoses in certain cases, which may not be feasible in all pathology settings. Lastly, the lack of molecular studies to correlate histopathological findings with viral subtypes represents a gap that future studies could address to strengthen the diagnostic framework. Additionally, interobserver reproducibility of the observed keratinocytic patterns was not assessed, and no formal scoring system or diagnostic sensitivity/specificity data were collected. These limitations highlight the need for future prospective studies to validate and refine these findings.

## Conclusions

This study highlights the diagnostic utility of atypical keratinocytic features in viral infections caused by HSV, VZV, and MC. In HSV/VZV, the recognition of nucleolar enlargement and early chromatin margination in keratinocytes adjacent to hallmark cytopathic changes serves as a valuable early diagnostic indicator. Similarly, the "pregnant seahorse appearance" of keratinocytes in MC provides a reliable clue for pathologists to perform deeper sections and confirm the diagnosis. These findings are observational and hypothesis-generating and should be interpreted within the context of a single-institution, retrospective case series. Future studies incorporating prospective case review, blinded interpretation, interobserver analysis, and correlation with molecular or virological data will be critical to validating the diagnostic reliability and clinical relevance of these keratinocytic features.
